# Posterior Approximate Clustering-Based Sensitivity Matrix Decomposition for Electrical Impedance Tomography

**DOI:** 10.3390/s24020333

**Published:** 2024-01-05

**Authors:** Zeying Wang, Yixuan Sun, Jiaqing Li

**Affiliations:** 1 School of Mechanical Engineering, University of Shanghai for Science and Technology, Shanghai 200093, China; 2 School of Energy and Power Engineering, University of Shanghai for Science and Technology, Shanghai 200093, China

**Keywords:** electrical impedance tomography (EIT), sensitivity matrix, *k*-means clustering, regularization method

## Abstract

This paper introduces a sensitivity matrix decomposition regularization (SMDR) method for electric impedance tomography (EIT). Using *k*-means clustering, the EIT-reconstructed image can be divided into four clusters, derived based on image features, representing posterior information. The sensitivity matrix is then decomposed into distinct work areas based on these clusters. The elimination of smooth edge effects is achieved through differentiation of the images from the decomposed sensitivity matrix and further post-processing reliant on image features. The algorithm ensures low computational complexity and avoids introducing extra parameters. Numerical simulations and experimental data verification highlight the effectiveness of SMDR. The proposed SMDR algorithm demonstrates higher accuracy and robustness compared to the typical Tikhonov regularization and the iterative penalty term-based regularization method (with an improvement of up to 0.1156 in correlation coefficient). Moreover, SMDR achieves a harmonious balance between image fidelity and sparsity, effectively addressing practical application requirements.

## 1. Introduction

Electrical impedance tomography (EIT) is an imaging technique that continuously reconstructs and visualizes the electrical conductivity distribution inside an object [1,2,3]. EIT has the advantages of safety, no radiation, fast response, portability, and low cost. It has received widespread attention and application in multiple fields including industrial measurement and biomedical detection, e.g., multi-phase flow visualization detection [4,5,6], bedside lung monitoring [7,8], and gesture capture [9,10].

EIT reconstruction is highly nonlinear and ill-posed due to the limited number of available observations. Given this constraint, the solution is extremely sensitive to small perturbations caused by measurement noise and modeling errors, resulting in inherently low spatial resolution and instability in the reconstructed images [11]. In order to address this problem, many traditional imaging algorithms have been developed, including direct sensitivity coefficient method [12], Landweber-type algorithms [13,14], gradient algorithms [15,16], Newton algorithms [17,18], regularization algorithms [19,20,21], etc. In recent years, owing to the outstanding ability to solve nonlinear problems, deep learning has received widespread attention from academics [22,23,24]. Despite the fact that the deep learning-based EIT method has good reconstruction accuracy and robustness, it is difficult to effectively reconstruct objects that differ significantly from the training set data. Likewise, unless the system architecture is upgraded, it is difficult to quickly embed the trained model into the existing EIT hardware structure.

The improved methods increase the calculation amount and time complexity of the algorithm to some extent, and may also introduce new non-adaptive parameters. The main challenge in developing EIT is to improve reconstruction accuracy and stability while satisfying rapidity requirements. Due to its effectiveness and rapidity in solving ill-posed problems, regularization has received an abundance of attention. A number of regularization methods have been developed, including factorization-based regularization [25], multiplicative Tikhonov regularization [26], total variation-based regularization [27], monotonicity-based regularization [28], and sparsity-based regularization [29] structures. Li et al. proposed an adaptive $L_{p}$ norm regularization form, which preserves geometric shapes and captures small objects [30]. In previous work, an advanced penalty term was proposed to optimize the regularization method and further improve the reconstruction accuracy [21]. These algorithms can provide stable solutions, but they cannot achieve an appropriate balance of accuracy, stability, and efficiency.

The sensitivity matrix is the fundamental component of EIT reconstruction, but it is typically derived from a uniformly distributed field rather than the actual situation, introducing deviations in imaging results. Correcting the sensitivity matrix using existing information has proven effective in enhancing reconstruction accuracy, as demonstrated by the use of a redundant sensitivity matrix for improved results [31]. Chen et al. enhanced the quality of the image by iteratively correcting the sensitivity matrix deviation, albeit at the expense of heightened computational demands [32]. The integration of two prior pieces of information for sensitivity matrix generation yields a well-balanced and robust performance, but the observed improvement in reconstruction results is not substantial [33]. Ding et al. improved the spatial resolution of the image by considering the typically ignored second-order structure of the sensitivity matrix, yet this improvement is accompanied by limitations in robustness [34]. These studies underscore the pivotal role of the sensitivity matrix in EIT reconstruction accuracy, revealing the challenge of balancing algorithm complexity, robustness, and accuracy.

In this work, a reconstruction method based on posterior approximated sensitivity matrix decomposition is proposed for EIT reconstruction. Following regularized imaging, the image is divided into four clusters using the *k*-means clustering method, which are defined as object, object–artifact mixture, artifact, and background. Focusing on specific work areas corresponding to these clusters, the sensitivity matrix is decomposed. The new sensitivity matrix is then employed for re-reconstruction using a difference framework to achieve a more precise EIT image. Comparative analysis with typical Tikhonov regularization and a recently proposed iterative penalty algorithm demonstrate the superior accuracy of the proposed method. The presented work introduces a novel and effective approach in SMDR, offering advancements in accuracy and robustness in EIT reconstruction. Moreover, it demonstrates its adaptability to both continuous and discrete manifolds, and the smooth edge effect around the object is significantly eliminated.

The rest of this paper is structured as follows. Section 2 includes the description of the EIT mathematical model, the analysis of EIT image features, the description of the proposed algorithm, and the configuration of the experiments. Section 3 describes the results of simulation experiments and tank experimental imaging. Section 4 discusses the results. Finally, conclusions are drawn in Section 5.

## 2. Methods

### 2.1. Mathematical Model

**Figure 1:**
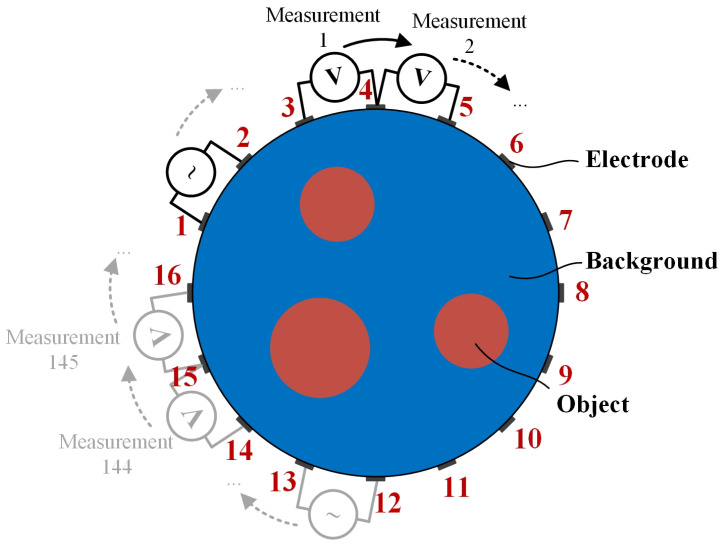
Electrical impedance tomography (EIT) detection principle.

EIT aims to reconstruct the conductivity distribution within a domain Ω. Figure 1 illustrates a typical 16-electrode sensing model, where the electrodes are evenly distributed around an observed object. A round of alternating current is injected into each adjacent electrode pair, and the voltage between the remaining pairs can be collected. After a complete measurement cycle, a boundary voltage vector with $N=n_{E}\times \left(n_{E}-3\right)$ elements can be obtained from $n_{E}$ electrodes, where half of the elements are independent.

The forward problem calculates the electric potential and the boundary voltages with known conductivity distribution and specified excitation pattern. This process can be formulated as

(1) U=F(σ,I)+e

where operator F(·) maps the inner conductivity distribution σ and injected current **I** into boundary voltage vector **U**; and **e** is the measurement noise. Such problem can be solved by numerical methods, e.g., the finite element method (FEM) [35].

The observation domain is partitioned into *M* discrete pixels. The conductivity distribution $\sigma \in R^{M\times 1}$ and the corresponding measurement sequence $U\in R^{N\times 1}$ can be linearly approximated as

(2) U=Sσ

where $S\in R^{M\times N}$ is sensitivity matrix, which is a Jacobin matrix linearizing the measurement U at a conductivity distribution σ. Each element in S represents the sensitivity of the conductivity of each discrete pixel to each measurement modality.

Assuming that the theoretical sensitivity matrix S is known, Equation (2) can be transferred as

(3) $$U-U_{0}=S\cdot \sigma -S_{0}\cdot \sigma_{0}$$

where $U_{0}$ represents the voltage vector of the uniform conductivity distribution $\sigma_{0}$, and $S_{0}$ can be calculated by the Lehr method [36] expressed as

(4) $${\left[S_{0}\right]}_{i,j}=\frac{\partial {\left[U_{0}\right]}_{i}}{\partial {\left[\sigma_{0}\right]}_{j}}$$

where i=1,2,…,M and j=1,2,…,N; ${\left[U_{0}\right]}_{i}$ represents the *i*th element of $U_{0}$; ${\left[\sigma_{0}\right]}_{j}$ represents the *j*th element of $\sigma_{0}$; and ${\left[J\right]}_{i,j}$ is the sensitivity coefficient of the *i*th measurement to the *j*th pixel. The Jacobin matrix **J** is often computed by FEM and depends on the shape of domain, background conductivity distribution, electrode position, and measurement strategy.

Equation (3) can be rewritten as

(5) $$\Delta U=\Delta S\sigma +S_{0}\Delta \sigma$$

where $\Delta U=U-U_{0}$, $\Delta \sigma =\sigma -\sigma_{0}$, and $\Delta S=S-S_{0}$ that can be ignored when Δσ is small. S is unavailable since σ is unknown; thus, the inverse problem is usually approximately solved by

(6) $$\Delta U\approx S_{0}\Delta \sigma$$

The difference conductivity distribution Δσ can be estimated with known difference voltage vector ΔU and sensitivity matrix $S_{0}$. Such process is referred to as inverse problem, which is a severely ill-posed problem, leading to unreliable and unstable solutions. Typically, regularization-based algorithms have been developed to reduce the instability.

The objective function of the regularization algorithm can be expressed as

(7) $$\Delta \sigma ={\arg\min}_{\Delta \sigma \in R^{M\times 1}}\left\{|\right|S_{0}\Delta \sigma -{\Delta U||}_{2}^{2}+{\lambda ||\Delta \sigma ||}_{2}^{2}\}$$

where the second term is the penalty function and λ is the regularization parameter, balancing the regularization and the penalty term.

The solution of Equation (7) can be estimated as

(8) $$\Delta \tilde{\sigma }=R\left(\lambda ,S_{0}\right)\Delta U$$

where $R(\lambda ,S_{0})$ represents the reconstruction matrix with regularization parameter λ and sensitivity matrix $S_{0}$. The typical Tikhonov-type regularization has been widely applied to seek such estimation, which uses an analytic expression as

(9) $$\Delta \tilde{\sigma }={\left(S_{0}^{T}S_{0}+\lambda L^{T}L\right)}^{-1}S_{0}^{T}\Delta U$$

where **L** is the regularization matrix and typically set as a unit matrix.

The reconstructed objects generated by such regularization method often suffer from excessive blur artifacts, resulting in low spatial resolution. It has been verified that, if the working area on the sensitivity matrix is adjusted to approximate the reconstruction object area, more precise results can be obtained. Such sensitivity matrix can be approximately generated by setting the object area as the working area of $S_{0}$ as

(10) $$S=S_{0}\left(\Delta \sigma E\right)$$

where $E\in R^{M\times M}$ is a unit matrix.

However, object area is the result that needs to be solved and is not available before the reconstruction process. Once the object area can be approximated, the approximated sensitivity matrix can be obtained and the accuracy of the reconstruction can then expected to be improved.

### 2.2. EIT Image Features

EIT reconstructed images are usually with low accuracy, as specifically shown in the (1) reconstructed object size error, (2) blur boundary, and (3) large artifact area. In previous work, pixels on one EIT image were divided into three clusters. However, when compared to the model, some pixels that should be objects are classified as artifacts based on the reconstructed conductivity values.

**Figure 2:**
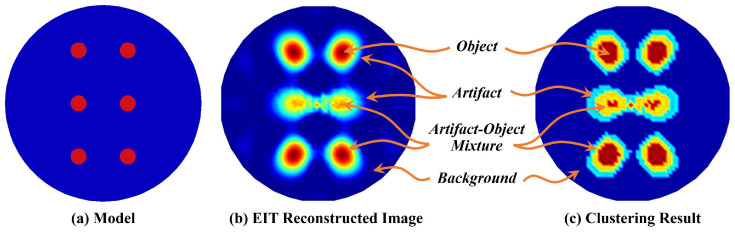
EIT reconstructed image and its *k*-means clustering result.

Figure 2a illustrates a model built on simulation platform. Figure 2b illustrates the EIT image reconstructed by typical regularization method. It can be found that there are roughly four types of pixels in the image, as follows:

1. *Object* pixels have the largest average reconstructed value, but the size has errors compared with the model. The closer to the center of the observed domain, the greater the size error.
2. *Artifact–Object Mixture* pixels correspond to the blurred part of the object boundary, and some of them corresponding to the model may by due to the object or the background part.
3. *Artifact* pixels usually adhere to the objects or are discretely distributed in the background.
4. *Background* pixels often correspond to the background part of the model and have the lowest average reconstructed values.

In previous work, the pixels of EIT images were classified into three types for the purpose of evaluating image quality [37]. The real object pixels, in the other hand, may be classified as artifacts in the reconstructed image. In this work, the purpose is to calculate the area where objects are located; thus, the image is divided into four types as analyzed above. In this way, the real object pixels are classified as object or artifact–object mixture type of the reconstructed image.

Pixels on an EIT image are classified into different sets, and different classification cutoffs need to be considered for different reconstructed images. Since the clustering method is unsupervised and adaptive, it is suitable for rapid classification of pixels in an EIT image.

Typical *k*-means is adopted in this work, which aims to partition the observation $\left\{{\left[\Delta \tilde{\sigma }\right]}_{1},{\left[\Delta \tilde{\sigma }\right]}_{2},\ldots {\left[\Delta \tilde{\sigma }\right]}_{M}\right\}$ into *k* clusters. The objective is to minimize the within-cluster sum of squares as

(11) $$\min\sum_{j=1}^{k}\sum_{i=1}^{M}{\left[w\right]}_{i,j}{∥{\left[\Delta \tilde{\sigma }\right]}_{i}-{\left[\mu \right]}_{j}∥}^{2}$$

where ${\left[\mu \right]}_{j}$ is the cluster center, representing the average value of elements of the *j*th cluster; and ${\left[w\right]}_{i,j}$ is updated by

(12) $${\left[w\right]}_{i,j}=\left\{\begin{array}{cc} 1, & \mathrm{if}\;i\mathrm{th}\;\mathrm{element}\;\mathrm{belongs}\;\mathrm{to}\;j\mathrm{th}\;\mathrm{cluster}\\ 0, & \mathrm{otherwise} \end{array}\right.$$

The cluster center is updated by

(13) $${\left[\mu \right]}_{j}=\frac{\sum_{i=1}^{M}{\left[w\right]}_{i,j}{\left[\Delta \tilde{\sigma }\right]}_{i}}{\sum_{i=1}^{M}\sum_{i=1}^{M}{\left[w\right]}_{i,j}}$$

According to the above analysis, set *k* as 4. For each iteration, observation elements are assigned to the clusters with the nearest center, and centers are updated. The clustering stops when the assignments no longer change. Figure 2c illustrates the *k*-means clustering result of Figure 2b. It can be observed that the clustering result is basically consistent with the four pixel types. The four clusters are sorted from large to small according to the cluster centers, corresponding to pixel types of *Object*, *Artifact–Object Mixture*, *Artifact*, and *Background*.

### 2.3. Sensitivity Matrix Decomposition Re-Reconstruction

Typical algorithms can be used to generate EIT images, and the clustering classification results can be used as rough posterior information. If the sensitivity elements corresponding to the background area are precisely set to 0 or directly removed, a sensitivity matrix corresponding to the approximate object area can be obtained, and the reconstruction results using such a sensitivity matrix can be more accurate. This is equivalent to reducing the amount of calculation.

According to the clustering results of EIT, the sensitivity matrix corresponding to different areas of interest can be decomposed into four parts as

(14) $${\left[S_{j}\right]}_{n,m}=\left\{\begin{array}{cc} {\left[S_{0}\right]}_{n,m}, & \mathrm{if}\;m\mathrm{th}\;\mathrm{pixel}\;\mathrm{belongs}\;\mathrm{to}\;j\mathrm{th}\;\mathrm{cluster}\\ 0, & \mathrm{otherwise} \end{array}\right.$$

where $\left[S_{j}\right]$ represents decomposed sensitivity matrix for *j*th cluster; j=1,2,3,4 represents the clusters of *Object*, *Artifact–Object Mixture*, *Artifact*, and *Background*, respectively.

The *Object* and the *Artifact–Object Mixture*, according to the EIT image feature analysis, can basically cover the real object area. A more accurate image can be re-reconstructed if these two areas are assigned as working areas, where a sensitivity matrix $S_{1}+S_{2}$ is adopted to replace $S_{0}$. However, there is inherent boundary ambiguity using regularization methods, and the working area still covers non-actual object areas, so there are still blurred boundary artifacts around the re-reconstructed object. Such artifacts on the EIT image is represented by $\Delta {\tilde{\sigma }}_{\overset{⌢}{pq}}$, representing the blurred reconstructed results affected by *p*th and (*q* = *p* + 1)th adjacent clusters.

Equation (8) can be transferred as

(15) $$\Delta \tilde{\sigma }=R\left(\lambda ,\sum S_{j}\right)\Delta U=\Delta \sigma +\sum_{p=1}^{3}\Delta {\tilde{\sigma }}_{\overset{⌢}{pq}}$$

where $\sum S_{j}$ represents the sensitivity matrix corresponding to the working area.

Differential re-reconstruction is performed, aiming to mitigate the smooth edge effect and improve image quality and object delineation in the reconstructed EIT images. Specifically, the working area is set as *Object*, *Artifact–Object Mixture*, and *Artifact*, the reconstruction can be expressed as

(16) $$\Delta {\tilde{\sigma }}_{\left\{1,2,3\right\}}=R\left(\lambda ,\sum_{j=1}^{3}S_{j}\right)\Delta U\approx \Delta \sigma +\sum_{p=1}^{3}\Delta {\tilde{\sigma }}_{\overset{⌢}{pq}}$$

When the working area is set as merely *Artifact*, the reconstruction can be expressed as

(17) $$\Delta {\tilde{\sigma }}_{3}=R\left(\lambda ,S_{3}\right)\Delta U\approx \sum_{p=2}^{3}\Delta {\tilde{\sigma }}_{\overset{⌢}{pq}}$$

where there is no Δσ since the real object is not within the working area *Artifact*.

The re-reconstructed result is calculated through the difference Equations (16) and (17) as

(18) $$\Delta {\tilde{\sigma }}^{\prime }=\Delta {\tilde{\sigma }}_{\left\{1,2,3\right\}}-\Delta {\tilde{\sigma }}_{3}\approx \Delta \sigma +\Delta {\tilde{\sigma }}_{\overset{⌢}{12}}$$

where the effect of blurred boundaries can be relatively eliminated.

Furthermore, reconstructed result is filtered and normalized by $\Delta {\tilde{\sigma }}_{h}=h\left(\Delta \tilde{\sigma }\right)$ as

(19) $${\left[\Delta {\tilde{\sigma }}_{h}\right]}_{i}=\left\{\begin{array}{cc} \frac{{\left[\Delta {\tilde{\sigma }}^{\prime }\right]}_{i}}{\max(\Delta {\tilde{\sigma }}^{\prime })}, & {\left[\Delta {\tilde{\sigma }}^{\prime }\right]}_{i}>0\\ 0, & \mathrm{otherwise} \end{array}\right.$$

where $\max(\Delta {\tilde{\sigma }}^{\prime })$ represents the maximum value in $\Delta {\tilde{\sigma }}^{\prime }$.

In order to further eliminate the residual artifacts, as post-processing, the reconstructed results are clustered again with *k* = 4 through Equations (11)–13), and the reconstruction elements in the *Artifact* and the *Background* area are directly set to 0 as

(20) $${\left[\Delta {\tilde{\sigma }}_{h}^{\prime }\right]}_{i}=\left\{\begin{array}{cc} {\left[\Delta {\tilde{\sigma }}_{h}\right]}_{i}, & \mathrm{if}\;i\mathrm{th}\;\mathrm{element}\;\mathrm{belongs}\;\mathrm{to}\;1\mathrm{th}\;\mathrm{or}\;2\mathrm{th}\;\mathrm{cluster}\\ 0, & \mathrm{otherwise} \end{array}\right.$$

Overall, the sensitivity matrix decomposition re-reconstruction (SMDR) algorithm is structured in Algorithm 1.
**Algorithm 1** SMDR Algorithm for EIT Reconstruction.**Input**: Voltage measurement: ΔU; Sensitivity matrix: $S_{0}$; Regularization parameter: λ.**Output**: Conductivity Vector: $\Delta {\tilde{\sigma }}_{h}^{\prime }$.**Begin**  1. Calculate the initial $\Delta \tilde{\sigma }$ by Equation (9);  2. Classify the pixels by Equations (11)–(13);  3. Decompose $S_{0}$ by Equation (14);  4. Calculate difference re-reconstructed image $\Delta {\tilde{\sigma }}^{\prime }$ by Equations (16)–(18);  5. Post-process $\Delta {\tilde{\sigma }}^{\prime }$ by filter Equation (19), clustering Equations (11)–(13), and clarity Equation (20).**End**

## 3. Experiments and Results

### 3.1. Experiment Configuration

A number of simulated phantoms were used to investigate the accuracy and robustness of the proposed algorithm. At the same time, typical Tikhonov-type (TK) regularization and iterative penalty term-based regularization (IPTR) were adopted to compare the proposed algorithm.

**Figure 3:**
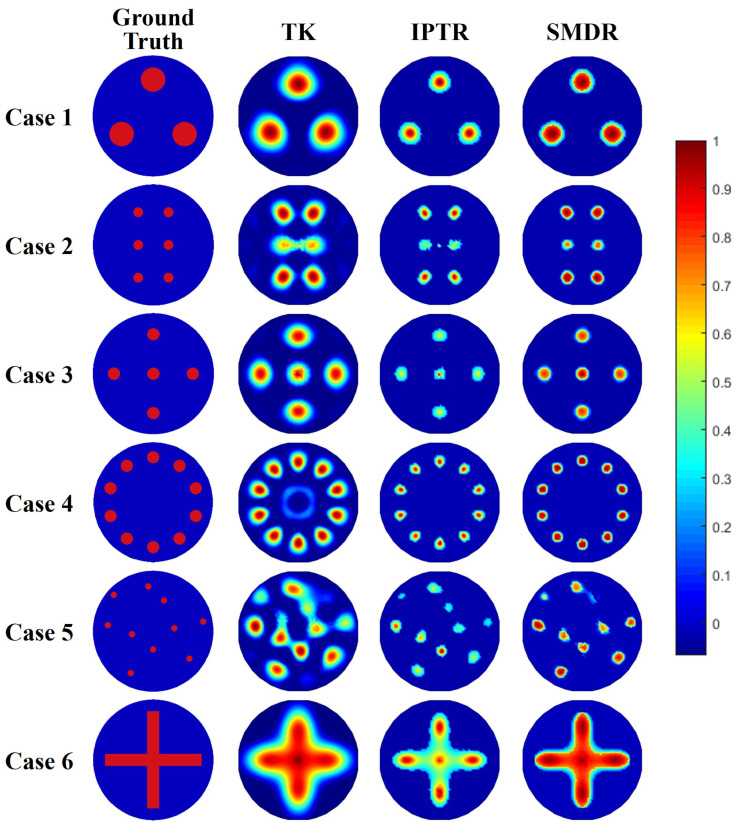
Reconstruction results of simulation models under different algorithms: TK, IPTR, and SMDR.

(1) Data

(a) Simulation data: A typical circular sensor with N=16 electrodes was built on COMSOL Multiphysics, as shown in Figure 1, where the radius of the circle observed domain is 1 m and the width of each electrode is 5 cm. The adjacent sensing strategy was adopted, as described in Figure 1, where each data frame contains N(N−3)=208 measured values. Reference voltage vector and sensitivity matrix were calculated from a homogeneous domain with conductivity of 1 S/m. Six phantoms within the circular sensor were constructed with object conductivity value at 2 S/m and background at 1 S/m. The conductivity distributions of six models are illustrated in the first column of Figure 3. To evaluate the robustness of the proposed algorithm, the signal-independent zero-mean additive white Gaussian noise was added to the measurement frame as

(21) $$\Delta U^{\prime }=\Delta U+n\left(\Delta U,SNR\right)$$

where n(ΔU,SNR) represents the noise of ΔU with a specific signal-to-noise (SNR) ratio. EIT data with no noise and SNR=30,40,50,55,60,65,70 were collected and applied to reconstruct EIT images, respectively, with the aim to evaluate the effectiveness and robustness of the proposed algorithm.

(b) Tank experiment data: Static tank experiments have been carried out in previous work [21]. Data were collected by the TJU-EIT system under adjacent measurement strategy, using the injection current of 5 mA and frequency of 100 kHz. The system consists of a PC, an FPGA-based digital hardware system, and a tank installed with a 16-electrode sensor system. The cylindrical tank has an inner diameter of 16 cm, a depth of 17 cm, and the electrode is horizontally 6 cm from the bottom. Plastic rods were employed as objects, the conductivity of which can be approximated as 0 S/m. The cylindrical tank was filled with saline with a conductivity of 0.01 S/m as the conductive background.

(2) Reconstruction and Evaluation

Reconstruction and numerical evaluation were performed on MATLAB 2020 for PC. Each EIT image is partitioned into 64×64 pixels. In this work, proposed SMDR based on the regularization algorithm was employed in EIT reconstruction. As a comparison, typical TK regularization and the IPTR algorithm were adopted. The parameter λ for each reconstruction was determined by unsupervised optimization [37]. Validation experiments were performed on all sets of EIT data using all mentioned reconstruction algorithms.

Furthermore, EIT suffers from low sensitivity in the central area of the observed domain. All reconstructed results are adjusted by the location compensation function as

(22) $$l\left({\left[\Delta \tilde{\sigma }\right]}_{i}\right)=\left(r-d_{i}\right){\left[\Delta \tilde{\sigma }\right]}_{i}$$

where *r* represents the radius of the observed domain and $d_{i}$ represents the distance between *i*th pixel and the center of observed domain.

Especially, in simulations, the realistic conductivity distribution is known. The reconstructed image can be evaluated by numerical evaluation metrics correlation coefficient and relative image error [38], expressed as

(23) $$CC=\frac{\sum_{i=1}^{M}\left({\left[\Delta \tilde{\sigma }\right]}_{i}-\bar{\Delta \tilde{\sigma }}\right)\left({\left[\Delta \sigma \right]}_{i}-\bar{\Delta \sigma }\right)}{\sqrt{\sum_{i=1}^{M}{\left({\left[\Delta \tilde{\sigma }\right]}_{i}-\bar{\Delta \tilde{\sigma }}\right)}^{2}\sum_{i=1}^{M}{\left({\left[\Delta \sigma \right]}_{i}-\bar{\Delta \sigma }\right)}^{2}}}$$

and

(24) $$RE=\frac{∥\Delta \tilde{\sigma }-\Delta \sigma ∥}{∥\Delta \sigma ∥}$$

where $\Delta \tilde{\sigma }$ and Δσ are the reconstructed conductivity variation and the realistic conductivity variation, respectively, ${\left[\Delta \tilde{\sigma }\right]}_{i}$ and ${\left[\Delta \sigma \right]}_{i}$ are the *i*th elements of $\Delta \tilde{\sigma }$ and Δσ, respectively, $\bar{\Delta \tilde{\sigma }}$ and $\bar{\Delta \sigma }$ are the average values of $\Delta \tilde{\sigma }$ and Δσ, respectively. CC indicates the correlation between the reconstructed image and the corresponding realistic distribution. The larger the value of CC, the smaller the value of RE, and the better the image quality.

### 3.2. Simulation Results

In EIT, algorithm improvements mainly observe the following directions:

1. Spatial resolution is expected to be improved, especially the resolution in the central area of the observed domain.
2. Object size and shape are expected to be recovered, and the reconstructed size of the same size object at different positions is expected to remain unchanged.
3. Artifact size is expected to be as small as possible, and the object boundary needs to be clear.

Figure 3 illustrates the reconstruction results of six models using three algorithms in simulation. As can be seen in the figure, TK regularization performs the worst overall because it oversmooths the image and has large sticking artifacts, as analyzed in Section 2.2. At the same time, in Case 2, the two objects near the center of the domain are significantly deformed, and the center resolution is poor.

The IPTR algorithm can reconstruct objects with overall more accurate size, but the reconstructed object values are uneven and the object shape has slight deformation. Especially in Case 1, there is an obvious local maximum area for each object. In Case 4, although the object size on the IPTR image is relatively accurate, circular objects are all slightly deformed into the shape of a water drop.

The proposed SMDR algorithm can accurately capture all objects of six cases. Overall, the SMDR algorithm can effectively remove discrete artifacts and object sticking artifacts, and the object boundaries are clearer than those reconstructed by the other algorithms. Although it is difficult for EIT to balance the reconstruction sizes of objects with different center distances, objects in the central area in Cases 2, 3, and 5 can be reconstructed more clearly, especially in terms of the consistency of the size and shape of the objects in each case which are all better preserved. In Case 5, all algorithms can identify the number of objects, but it is difficult to recover the size of all objects at the same time. SMDR basically recovers eight out of ten objects and maintains the size consistency of these eight objects, whereas the size consistency of the objects reconstructed using the other two algorithms is relatively poor. Regularization-based algorithms usually make the edges of the reconstructed objects smoother, making it difficult to recover right angles. In Case 6, although it is difficult for SMDR to reconstruct the right angles, SMDR retains the object area most accurately among the three methods.

**Figure 4:**
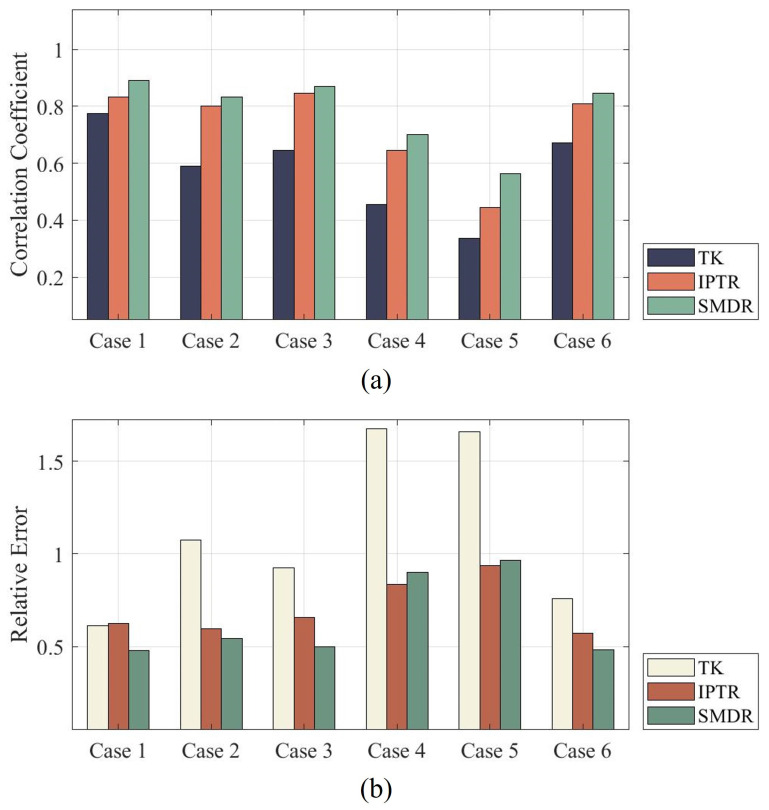
Numerical evaluation metric results of reconstructed images in Figure 3. (**a**) Results of Correlation Coefficient (CC). (**b**) Results of Relative Error (RE).

Figure 4 illustrates the CC and RE results of reconstructed images in Figure 3. Relatively consistent with image observation, the CC result of SMDR regularization is the highest among the three algorithms for each model. The RE result of SMDR regularization is the smallest in Cases 1, 2, 3, and 6, while there are slightly larger size and location biases on Cases 4 and 5 for SMDR, causing a slightly larger RE. Such numerical results indicate the worst imaging quality for TK, which is attributed to the artifacts of TK that reduce the accuracy of the reconstruction. For each model, SMDR has the largest CC, while the uneven distribution in IPTR objects causes the drop on CC. Overall, both image observation results and quality metrics verified the effectiveness and universality of SMDR.

Table 1 illustrates the computation time (in milliseconds) in Figure 3 to evaluate the computational efficiency of SMDR in comparison to the established algorithms. The average computation time for SMDR is approximately twice that of TK, as reflected in specific numerical values indicating SMDR with longer computation time. For instance, on average, SMDR takes 79.0 ms compared to TK with 36.2 ms. Despite this relative slowness compared to TK, SMDR consistently surpasses IPTR in terms of computation speed across all cases. It is important to note that prior research has demonstrated the feasibility of IPTR for real-time reconstruction. This highlights the efficiency of SMDR, effectively meeting the crucial requirement for swift EIT calculations.

**Figure 5:**
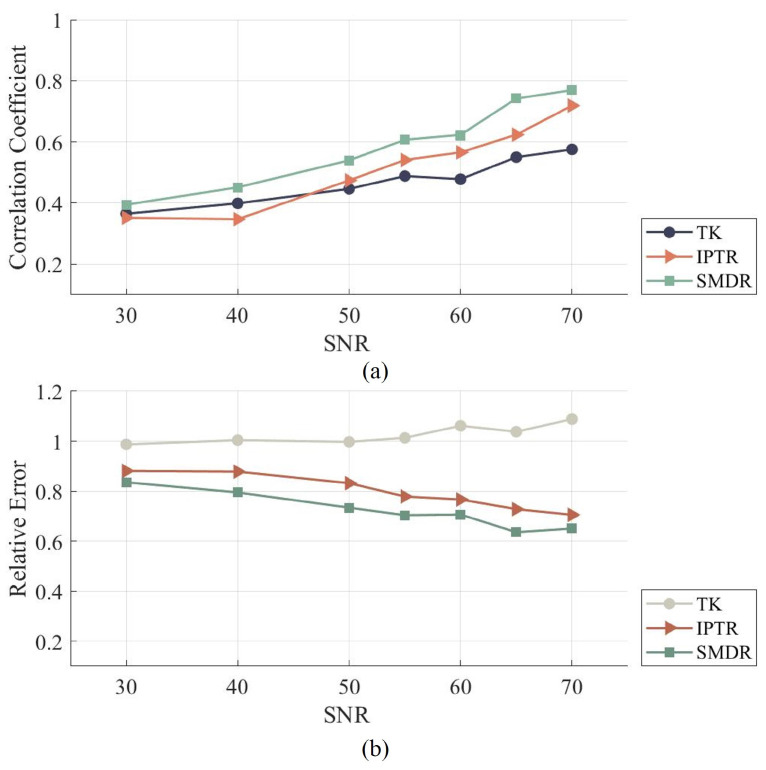
Average evaluation results under different SNR and using different algorithms: TK, IPTR, and SMDR. (**a**) Results of correlation coefficient (CC). (**b**) Results of relative error (RE).

Figure 5 compares the effects of the three different algorithms under different SNR, where the average RE and CC of the multiple Cases under different SNRs for each algorithms are listed. It can be seen that SMDR always shows optimal performance under various noises. Under different noises, the CC value of SMDR is higher than that of the other two algorithms, and its RE value is lower than for the other two algorithms, which indicates that it has higher reconstruction accuracy. It can be concluded that the SMDR algorithm has good robustness and can maintain stable performance in different noise environments.

### 3.3. Tank Experimental Results

**Figure 6:**
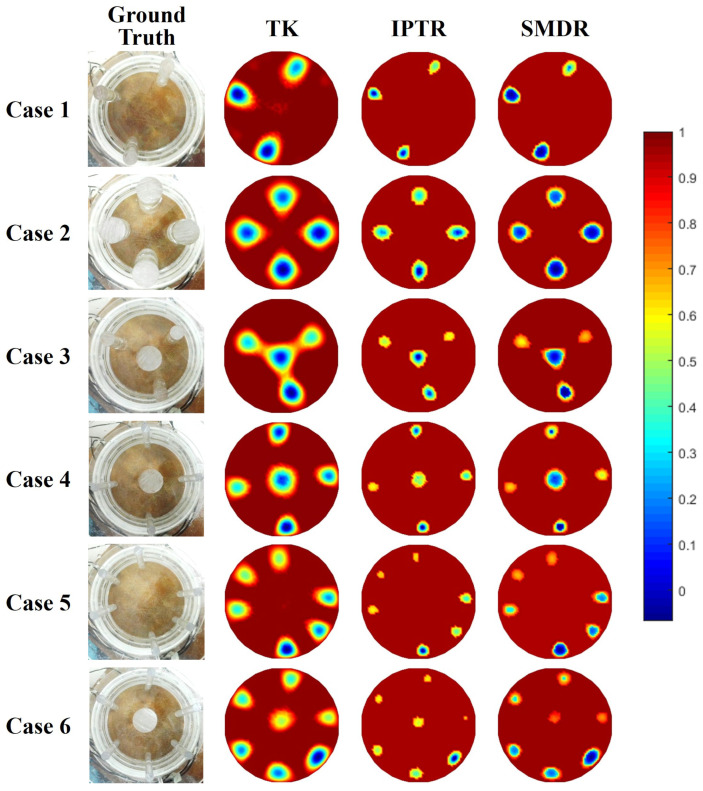
Reconstructed images in experiments under different algorithms: TK, IPTR, and SMDR.

The real distribution and reconstructed images are illustrated in Figure 6. It can be concluded that the three EIT algorithms can reconstruct all objects. However, TK-reconstructed images suffer from the largest artifacts. Especially in Case 3, all objects are stuck together by the surrounding blur artifacts. Additionally, TK has difficulty maintaining the shape of an object when it is close to the domain boundary. There is significant deformation of the circular objects in Cases 1, 2, and 5. At the same time, when the objects are small (in Cases 1, 4, 5, and 6), the reconstructed objects in TK are significantly larger than the real objects. In contrast, images reconstructed using IPTR are virtually free of artifacts. However, IPTR still slightly over-penalizes the solution, and the dimensions of all objects are still smaller than those of real objects.

For the proposed SMDR algorithm, the location and size of the objects can be reconstructed relatively accurately with almost no artifacts. Unfortunately, the dimensions of the central circular object in Model 6 are difficult to recover due to having multiple objects close to the domain edge.

**Figure 7:**
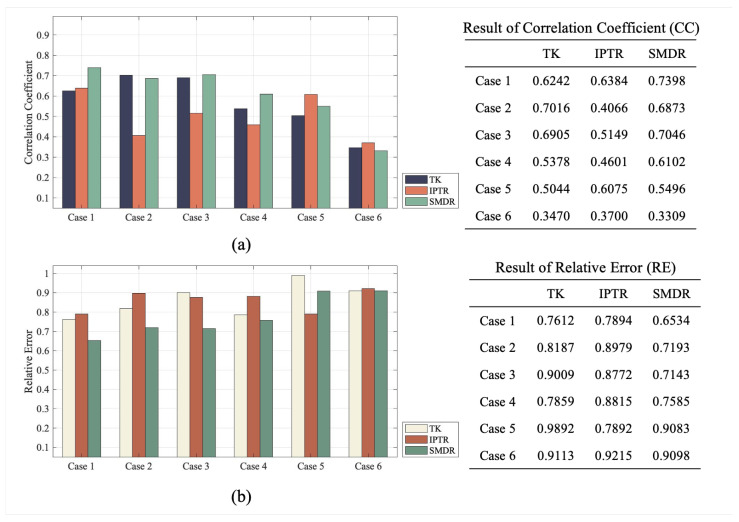
Numerical evaluation metric results of reconstructed images in Figure 6. (**a**) Results of Correlation coefficient (CC). (**b**) Results of relative error (RE).

In examining the performance across various models, the SMDR almost consistently demonstrates notable advantages. Figure 7 illustrates the CC and RE results of reconstructed images in Figure 6. In Case 1, SMDR achieves a substantial improvement with a CC value of 0.7398 compared to TK in 0.6242 and IPTR in 0.6384. This trend continues in Case 2, where SMDR exhibits comparable accuracy to TK but outperforms IPTR. Similarly, in Cases 3 and 4, SMDR surpasses both TK and IPTR with CC values. While SMDR demonstrates consistent advantages across multiple cases, it is crucial to note a specific instance where its performance is less favorable. In Case 6, SMDR exhibits a worse performance. This can be attributed to the erroneous reconstruction of a larger target in the center of the field. This discrepancy is a result of inaccuracies in the central area, which impact the reconstruction process adversely.

Analyzing the results of RE, it is evident that SMDR consistently achieves the smallest values across most cases, indicating superior performance in accuracy compared to TK and IPTR. Overall, the proposed SMDR algorithm has relatively high fidelity in terms of reconstruction, improving the quality of the reconstructed images compared with traditional algorithms.

## 4. Discussion

A sensitivity matrix decomposition re-reconstruction algorithm is proposed in this paper. The reconstruction process involves utilizing *k*-means cluster classification of the preliminary imaging solution as an approximate posterior. Subsequently, the sensitivity matrix is decomposed based on the classification result, followed by substituting the sensitivity matrix into the difference method for re-reconstruction. Finally, a second clustering step is employed to eliminate artifacts in the post-processing of the EIT image. Through simulation experiments and the comparison of typical regularization algorithms, the recently proposed iterative penalty with improved penalty term (IPTR) regularization algorithm, and the proposed sensitivity matrix decomposition regularization (SMDR) method using experimental data were conducted. The results demonstrate the effectiveness of the SMDR algorithm in enhancing EIT imaging quality, object fidelity, and robustness.

Spatial resolution: SMDR is capable of reconstructing both discrete and continuous objects. At the same time, the recognition ability of the central area has been improved, and the object reconstruction results in the central area are clearer. In the same reconstruction object, the values of the reconstructed solution are relatively uniform, and there is relatively no local maximum.

Object size and shape: SMDR has the ability to better reconstruct the size and shape of the object. Objects of the same size within the observed domain exhibit minimal deviation and slight deformation in the reconstructed SMDR object, showcasing fidelity in both shape and size. Experimental data verification indicates that SMDR reconstructs smaller object sizes in the central area compared to traditional methods that tend to enlarge objects. In future work, fusion algorithms will be considered to address object size inaccuracies.

Artifact size: To address the smooth edge effect, the proposed algorithm employs a dual strategy. Firstly, it decomposes sensitivity based on image features and performs differential reconstruction to accurately eliminate mixed artifacts from the initial coarse imaging. Secondly, post-processing of the re-reconstructed image is carried out to further identify and eliminate edge artifacts. In comparison with typical regularization, the re-reconstruction performed by SMDR effectively reduces blur artifacts around objects. This highlights the ability of SMDR to balance artifact reduction while sharpening object edges.

Algorithm: The SMDR algorithm stands out for its parameter management. It avoids introducing indeterminate parameters, ensuring adaptability and effectiveness in EIT adaptive imaging detection. This adaptive nature contributes to its applicability across various scenarios without introducing ambiguous parameters.

Limitations: SMDR demonstrates notable advantages in specific electrical impedance tomography scenarios, where its effectiveness stands out. However, its performance across diverse domains and in comparison to traditional or emerging techniques may exhibit variations. Moreover, the current validation of SMDR primarily centers on scenarios featuring two-phase fields. The applicability of SMDR may encounter limitations when confronted with intricate variations in conductivity. Furthermore, although SMDR exhibits improved capability in identifying sharp right-angle edges, its optimal performance is still more suited for recognizing objects with edges characterized by convex circular arcs.

## 5. Conclusions

An advanced method was developed to improve accuracy and robustness for EIT reconstruction. The *k*-means clustering is the prerequisite operation for sensitivity matrix decomposition. The quantitative evaluation on the simulation platform and experimental data reveals compelling improvements in accuracy and robustness. For instance, the proposed method demonstrated a significant increase in correlation coefficient, reaching an up to 0.1156 improvement compared to conventional regularization. Additionally, a notable reduction in relative image error was observed (up to 0.1866), underscoring the enhanced fidelity achieved by the method. These quantitative scores provide the substantial advancements brought about by the proposed approach.

Despite advancements, specific challenges require attention in future research. Notably, reconstruction performance in scenarios with complex geometries and irregular object shapes poses a crucial hurdle for applicability across diverse real-world scenarios. Another challenge involves reconstructing multiphase distribution fields accurately, essential for expanding utility to applications like medical imaging and industrial processes. Addressing and strategically tackling these challenges will guide subsequent studies in refining and expanding the capability of the proposed EIT reconstruction.

Subsequent studies should focus on exploring method adaptability to dynamic scenarios (e.g., physiological changes, fluid dynamics) and assess performance in resource-limited environments. These targeted investigations ensure purposeful research, fostering the ongoing innovation and refinement of EIT reconstruction methods.

## Figures and Tables

**Table 1:** Calculation time of reconstruction in Figure 3 with different algorithms.

Algorithm	TK (ms)	IPTR (ms)	SMDR (ms)
Case 1	38.9	109.1	72.4
Case 2	33.3	108.0	93.1
Case 3	40.8	127.4	77.5
Case 4	31.1	112.7	72.1
Case 5	32.5	130.5	69.5
Case 6	41.0	111.7	89.1
Average	36.2	116.6	79.0

## Data Availability

The raw data supporting the conclusions of this article will be made available by the authors on reasonable request.
